# Cross‐Cultural Adaptation of the Clinical Frailty Scale for Critically Ill Patients in Spain and Concurrent Validity With FRAIL‐Es

**DOI:** 10.1002/nop2.70064

**Published:** 2025-02-17

**Authors:** Susana Arias‐Rivera, María Nieves Moro‐Tejedor, Fernando Frutos‐Vivar, Cristina Andreu‐Vázquez, Israel John Thuissard‐Vasallo, María Mar Sánchez‐Sánchez, Raquel Sánchez‐Izquierdo, Lorena Oteiza‐López, Sonia López‐Cuenca, Marta Checa‐López, Raquel Jareño‐Collado, Virginia López‐López, Eva Isabel Sánchez‐Muñoz, Luis Fernando Carrasco Rodríguez‐Rey, María Jesús Frade‐Mera, Irene Cortés‐Puch, Rebeca Padilla‐Peinado, Alejandro Huete‐García, Amanda Lesmes‐González Aledo, Federico Gordo‐Vidal, Ana Rodríguez‐Merino, Mónica Vázquez‐Calatayud, Gloria Vázquez‐Grande, Dolores Mateo, Raquel Herrero‐Hernández, Marta Raurell‐Torredà

**Affiliations:** ^1^ Doctoral program in Nursing and Health University of Barcelona Barcelona Spain; ^2^ Department of Nursing Research Hospital Universitario de Getafe Getafe(Madrid) Spain; ^3^ Nursing Research Support Unit General University Hospital, Gregorio Marañón Madrid Spain; ^4^ Gregorio Marañon Health Research Institute (IiSGM) Madrid Spain; ^5^ Red Cross University School of Nursing Autonomous University of Madrid Madrid Spain; ^6^ Intensive Care Unit Hospital Universitario de Getafe Getafe (Madrid) Spain; ^7^ Departamento de Medicina Facultad de Ciencias Biomédicas y de la Salud, Universidad Europeade Madrid, Villaviciosa de Odó (Madrid) Madrid Spain; ^8^ Geriatric Department Hospital Universitario de Getafe Getafe (Madrid) Spain; ^9^ Critical Cardiology Care Unit Hospital Universitario 12 de Octubre Madrid Spain; ^10^ Department of Nursing Faculty of Nursing, Physiotherapy, and Podology, Complutense University of Madrid Madrid Spain; ^11^ Intensive Care Unit Hospital Universitario 12 Octubre Madrid Spain; ^12^ Division of Pulmonary Critical Care and Sleep Medicine, University of California Davis Medical Center Sarcamento EEUU USA; ^13^ Intensive Care Unit Hospital Virgen de la Salud Toloedo Spain; ^14^ Intensive Care Unit HM Hospitales Madrid Spain; ^15^ Intensive Care Unit Hospital Universitario del Henares Coslada (Madrid) Spain; ^16^ Grupo estable de investigaciónen Patología Crítica Facultad de Medicina, Universidad Francisco de Vitoria Madrid Spain; ^17^ Royal Brompton and Harefield Hospital Trust London UK; ^18^ Area of Nursing Professional Development Clínica Universidad de Navarra Pamplona Spain; ^19^ Faculty of Nursing University of Navarra Pamplona Spain; ^20^ Navarra Institute for Health Research (IdiSNA) Pamplona Spain; ^21^ Section of Critical Care Medicine Department of Medicine, University of Manitoba Winnipeg Canada; ^22^ Intensive Care Unit Broomfield Hospital, Mid Essex NHS Foundation Trust, Chelmsford Essex UK; ^23^ CIBER de Enfermedades Respiratorias Instituto de Investigación Carlos III Madrid Spain; ^24^ Departamento de Bioingeniería Universidad Carlos III Madrid Spain; ^25^ Facultad de Enfermería Universidad de Barcelona Barcelona Spain

**Keywords:** cross‐cultural comparison, frailty, intensive care units, validation studies

## Abstract

**Aims:**

To adapt the Clinical Frailty Scale (CFS) into Spanish and assess its concordance with the Spanish version of the FRAIL scale (FRAIL‐Es) in the context of intensive care.

**Design:**

Validation study of frailty assessment scales in critically ill patients.

**Methods:**

The study was conducted in two phases. The first phase consisted of translating, culturally adapting, and validating the CFS into Spanish. The second phase consisted of a metric descriptive study to assess the concurrent criterion validity of the adapted CFS with FRAIL‐Es in a cohort of intensive care patients. Both scales were assessed upon admission to intensive care and at 3, 6, 9, and 12 months post‐hospital discharge. Analysis was performed using *T*‐Student/Mann–Whitney, chi‐squared and Cohen's Kappa tests.

**Results:**

Successful adaptation of the CFS with minimal changes was achieved, demonstrating its applicability in the evaluated context. The pilot study indicated that CFS‐Es is easy to assess, but some subjectivity in interpretation was noted. CFS‐Es and FRAIL‐Es were applied to 212 patients, revealing variations in frailty prevalence. The concordance and correlation between the CFS and FRAIL scales are robust. These differences suggest that the choice of scale may impact the identification of frail patients. These results emphasise the importance of considering specific characteristics of each scale when assessing frailty in critically ill patients, providing valuable information for clinical implementation and research in this field.

**Patient or Public Contribution:**

Assessing frailty upon admission can be helpful in the care of frail patients, allowing the development of specific care plans based on pre‐existing frailty.

## 1 Introduction

The term ‘frailty’ is common in everyday language, but its application can vary according to different criteria. Socially, frailty is perceived as a condition of functional dependence in which the frail person requires assistance from others to survive (Gillick 1989). However, this notion is not limited solely to the social aspect, as various factors influence the degree of frailty, which is closely linked to the age‐related decline in homeostasis or the loss of physiological reserve (De Biasio et al. 2020). In addition to psychosocial and biomedical approaches (Rockwood et al. 1994), Witten (1985) proposed a dynamic model in which various factors interact, defining functional status based on the balance between these elements at each moment.

Numerous studies have linked frailty to mortality in older hospitalised patients (Muscedere et al. 2017). However, an increase in mortality has also been observed in frail patients under 65 years old (Bagshaw et al. 2016; Brummel et al. 2017; Hanlon et al. 2018). Frail individuals are more susceptible to stressors, such as new drugs, infections, or minor surgical procedures, leading to faster deterioration compared to non‐frail individuals (Abellan van Kan, Rolland, Morley, et al. 2008). Therefore, assessing frailty during patient admission can provide healthcare professionals with valuable information, enabling them to determine interventions that may be most beneficial for this particular group of patients, thus contributing to more precise and personalised care.

As mentioned above, various tools exist for assessing frailty from different perspectives (Buta et al. 2016). The Clinical Frailty Scale (CFS) has gained widespread acceptance due to its ease of use and the possibility of employing it during the acute phase of an illness, either directly with the patient or through interviews with family members (Pugh et al. 2018). This scale has been applied to a wide variety of populations, both in hospitalised and outpatient settings (Church et al. 2020). Clinicians frequently use it to tailor care plans according to individual needs, and researchers also utilise it in their investigation studies. In the field of intensive care, the CFS and the FRAIL scale (Abellan van Kan, Rolland, Bergman, et al. 2008; Abellan van Kan, Rolland, Morley, et al. 2008) are the most used frailty scales.

## 2 Background

The CFS was developed to summarise overall physical fitness or frailty in older adults after assessment by an expert clinician. This scale can predict the older adult's death or the need for institutional care (Rockwood et al. 2005). Initially introduced in 2005 as a 7‐level scale, validated in the Canadian Study of Health and Aging (CSHA) (Rockwood et al. 2005), two levels were added in 2007, ranging from 1 (very fit) to 9 (terminally ill). A patient is considered frail with a level of 5 or higher. This addition recognised that severely ill or terminally ill elderly individuals required different care plans and needed to be differentiated (Clinical Frailty Scale n.d.). In 2020, version 2.0 was published, providing guidance on using the scale and including revisions in naming each level (Rockwood and Theou 2020).

The FRAIL scale was developed in 2007 by the Geriatric Advisory Panel of the International Academy of Nutrition and Aging after conducting a thorough review of available assessment tools. These experts carefully determined which components should be included in a tool capable of detecting potential frailty syndrome. This scale allows subjects to be categorised into three groups: good health and absence of frailty (0 points), pre‐frailty (1–2 points) and frailty (3–5 points). The FRAIL scale has been adapted explicitly to Spanish for adult patients entering intensive care units (ICUs) (Arias‐Rivera et al. 2023).

It is crucial to understand that tools intended for use across various cultures must not only undergo linguistic translation but also conceptual adaptation. This adaptation is a significant step in making these tools applicable in different settings from where they were originally developed.

For the successful implementation of the CFS in critically ill patients in Spain, a comprehensive transcultural adaptation is not just a requirement but a thorough approach. Therefore, we performed a linguistic adaptation, as the original scale was developed in English, and we aimed to use a Spanish version of the scale. We also applied a cultural adaptation of the scale to a different country (Canada vs. Spain) and a clinical adaptation to a different population (elderly population vs. critically ill patients over 18 years old).

We have not found a cross‐cultural adaptation of the CFS in the literature for Spain. Therefore, we propose to develop the Spanish version of the CFS (CFS‐Spain).

The aims of this study are: (1) to outline the process of adapting a Spanish‐translated version of the CFS for use in critical care medicine for patients over 18 years old admitted to Intensive Care Units in Spain, and (2) to assess the agreement and relationship between this new Spanish adapted CFS and the Spanish version of the FRAIL scale (FRAIL‐Es) (Arias‐Rivera et al. 2023).

## 3 Methods

### 3.1 Study Design

The study comprised two phases, involving the translation, adaptation and validation of the CFS to Spanish, followed by a descriptive metric study to assess the concurrent criterion validity with the Spanish version of the FRAIL scale (FRAIL‐Es). The COSMIN checklist was followed during the process (Gagnier et al. 2021).

### 3.2 Data Tools

The CFS graphically describes various levels of frailty and disability, assigning scores ranging from 1 (very fit) to 9 (terminally ill). Individuals in the first three levels are considered non‐frail, those in level 4 are considered vulnerable or with very mild frailty (according to CFS 2.0), and individuals beyond the fourth level exhibit varying degrees of frailty. Level 9 includes individuals whose life expectancy is less than 6 months, although they may not be inherently frail. The CFS also has a specific classification for patients with dementia (Clinical Frailty Scale n.d.).

The FRAIL‐Es scale (Arias‐Rivera et al. 2023), like the original FRAIL scale (Abellan van Kan, Rolland, Bergman, et al. 2008; Abellan van Kan, Rolland, Morley, et al. 2008), encompasses five domains forming its acronym: Fatigue, Resistance (ability to climb 10 steps), Ambulation (ability to walk several 100 m), number of Illnesses (including hypertension, diabetes, cancer, chronic lung disease, heart attack, congestive heart failure, angina, asthma, arthritis, stroke and kidney disease) and unintentional loss of weight (> 5%). The scale scores range from 0 to 5 points based on the presence or absence of each item, categorising subjects into three groups: good health and absence of frailty (0 points), pre‐frailty (1–2 points) and frailty (3–5 points) (Abellan van Kan, Rolland, Morley, et al. 2008).

### 3.3 Phase 1: Cultural and Linguistic Adaptation of the CFS

The cultural adaptation and translation involved several stages, adhering to standardised criteria (Sousa and Rojjanasrirat 2011). Permission was obtained from the scale's authors (Data S1), who participated in the correlation between the original scale and the result of the back‐translation.

#### 3.3.1 Translation of the CFS Original and Generation of the First Version of the Clinical Frailty Scale‐España (CFS‐Es)

Considering that the scale will be applied by healthcare professionals in the intensive care units, the scale's translation into Spanish involved three bilingual healthcare professionals specialised in intensive care (two nurses and one physician). They were provided with the original version of the instrument along with an explanation of its characteristics, utility and the translation's objective.

#### 3.3.2 Back‐Translation of the CFS‐Es′ First Version to English

A committee of bilingual experts (two intensive care nurses, two intensivist physicians, one geriatric physician with expertise in frailty and one methodologist) unified the three Spanish versions, assessing their semantic equivalence. For the formation of the expert committee, intensive care nurses and physicians were selected, as they are the professionals who will ultimately apply the scale. A geriatrician specialising in frailty was included for her more conservative perspective on frailty scales, and a methodologist was involved for methodological support. Three Spanish intensivist physicians residing in the United Kingdom, the United States and Canada performed the back‐translation to English. The expert committee unified the resulting English versions.

#### 3.3.3 Agreement Between the Two English Scales, the Original and the One Obtained After Back‐Translation

The scale authors compared the unified version from back‐translations with the original scale to assess semantic, technical and conceptual equivalence. The Spanish version was modified based on the author's feedback, resulting in the second version of the CFS‐Es.

#### 3.3.4 Pilot Test and Generation of the Spanish Version of the CFS (CFS‐Es)

Ten intensive care professionals (5 nurses with over 10 years of experience in the ICU and 5 intensivist physicians) applied the second version of the CFS‐Es to 30 critical patients with different age ranges (< 50 years, 50–65 years and > 65 years). The objective was to assess the relevance, using a 4‐point Likert scale (from 1 not relevant to 4 very relevant), and the comprehensibility (good, acceptable or poor) of each item. Each professional recorded the time it took to implement the scale for each evaluated patient. Following discussion within the expert committee and the incorporation of professional feedback, the third and final version of the CFS‐Es was reached.

### 3.4 Phase 2: Analysis of Criterion Validity Between the Spanish Versions of CFS and FRAIL‐Es Scale

Following the adaptation process, the final version of the CFS‐Es and the FRAIL‐Es was implemented in ICU patients at admission and 3, 6, 9 and 12 months post‐hospital discharge.

#### 3.4.1 Participants

Inclusion criteria included adult patients with ICU stays lasting more than 48 h, excluding only those with COVID‐19 or suspected of impending death. The participants' sex and age were recorded.

#### 3.4.2 Data Collection

All evaluations were conducted by research team members, with the same investigator assessing patients using both scales simultaneously.

The principal investigator conducted the baseline assessment through face‐to‐face interviews with patients or their closest relatives if the patient was non‐communicative. This assessment reflected the patient's condition 1 month before hospitalisation. Two research team members conducted follow‐up assessments at 3, 6, 9 and 12 months through telephone interviews with the patient or their closest relatives. Both members of the research team were familiar with the scale, but they did not apply it in their clinical practice or receive training for its implementation.

### 3.5 Statistical Analysis

Quantitative variables were presented as medians with interquartile range (IQR) or means with standard deviation (SD), and categorical variables were expressed as absolute (*n*) and relative (%) frequencies. Normality was assessed using the Shapiro–Wilk test. The *T*‐Student or Mann–Whitney *U*‐test was used to compare quantitative variables (according to parametric behaviour), and the chi‐squared test was used for categorical variables.

Concordance between CFS‐Es and FRAIL‐Es was assessed using Cohen's Kappa, considering two groups (non‐frail patients—CFS‐Es 1–4 and FRAIL‐Es 0–2—and frail patients—CFS‐Es 5–9 and FRAIL‐Es 3–5) and three groups (non‐frail patients—CFS‐Es 1–3 and FRAIL‐Es 0, vulnerable or pre‐frail patients—CFS‐Es 4 and FRAIL‐Es 1–2, and frail patients—CFS‐Es 5–9 and FRAIL‐Es 3–5). Additionally, considering that at level 4 of the CFS 2.0 the patients are classified with very mild frailty (previously referred to as vulnerable in earlier versions), concordance with the FRAIL‐Es has also been assessed, considering patients rated with CFS‐Es 4–9 as frail. Kappa values less than 0.2 were considered insignificant agreement, 0.2–0.4 as low agreement, 0.4–0.6 as moderate agreement, 0.6–0.8 as good agreement and greater than 0.8 as very good agreement (Cohen 1960).

The correlation between CFS‐Es and FRAIL‐Es was assessed using Pearson's correlation coefficient after checking for the parametric behaviour of variables. A null correlation was considered for values below 0.10, weak for levels ≤ 0.10–0.30, moderate for levels between ≤ 0.30–0.50 and strong for values ≥ 0.50 (Hernández Lalinde et al. 2018).

A two‐tailed *p*‐value of < 0.05 was considered statistically significant. Statistical analyses were conducted using IBM SPSS Statistics for Windows (version 29.0, IBM Corp., Armonk, NY).

### 3.6 Ethics

The study protocol underwent review and approval by the Ethics Committee for Drug Research (CEIm) of the Hospital (IRB number: CEIm19/42CEIm19/42), which adds rigour to the study (Cathala and Moorley 2018). Patient consent was obtained for inclusion in the study, either directly from the patient or from their closest family member in cases where the patient was unable to provide personal consent. Each patient was assigned an alphanumeric code known only to the study's principal investigator to preserve the confidentiality of their data.

## 4 Results

### 4.1 Translation, Back‐Translation and Concordance

The original scale authors considered good agreement for all items but requested minor changes in level 6 of the scale (for patients with and without dementia). After retaining ‘cuing, standby’, as in the original, rather than just ‘standby’ as in the back‐translated version, the original scale authors accepted the scale. This change led to modifying the first version of the CFS‐Es, adding ‘stimulation, accompaniment’ instead of just ‘accompaniment’ in level 6 of the second version of the CFS‐Es.

### 4.2 Clinical Pilot

The overall assessment of the scale was good among all professionals. However, due to the absence of closed‐ended questions to specify the patient's frailty level, the assessment depended on the interviewer's expertise, leading to high subjectivity. The scale's median (IQR) application time was 3 min and 10 s (1–7 min). Nurses took less time than physicians, although the differences were not significant (Median [IQR]: 2 min and 9 s [1 min–4 min and 47 s] vs. 5 min [1–10 min]; *p* = 0.183).

**FIGURE 1:**
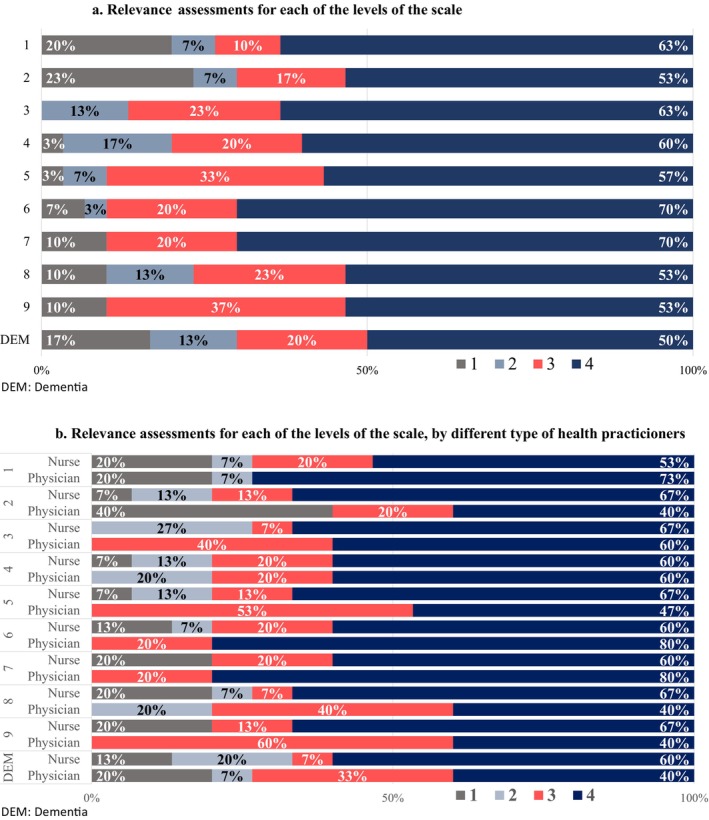
(a) Relevance assessments for each of the levels of the scale. (b) Relevance assessmfents for each of the levels of the scale, by different type of health practitioners.

Nurses and physicians assessed the relevance of each level similarly (Table 1), with over 50% of the evaluators assigning the maximum relevance score (4 points) to each level (Figure 1a). While most nurses rated all levels as highly relevant with 4 points (Figure 1b), physicians considered levels 2, 5, 8 and 9 and the evaluation of dementia less relevant. The least relevant level was level 2 (40% relevance 1), as some comorbidities could contribute to frailty despite the patient being active. Physicians also found levels 8 and 9 less relevant due to little difference between them, as both cases involved patients nearing the end of life, and the level of dependence seemed insignificant for differentiation.

**TABLE 1:** Assessment of the relevance of each level of the scale.

Level	Global *N* = 30	Nurses *N* = 15	Physicians *N* = 15	*p*
1, median (IQR)	4 (2–4)	4 (2–4)	4 (2–4)	0.512
2, median (IQR)	4 (2–4)	4 (3–4)	3 (1–4)	0.126
3, median (IQR)	4 (3–4)	4 (2–4)	4 (3–4)	0.870
4, median (IQR)	4 (3–4)	4 (3–4)	4 (3–4)	0.967
5, median (IQR)	4 (3–4)	4 (3–4)	3 (3–4)	0.683
6, median (IQR)	4 (3–4)	4 (3–4)	4 (4–4)	0.267
7, median (IQR)	4 (3–4)	4 (3–4)	4 (4–4)	0.267
8, median (IQR)	4 (3–4)	4 (2–4)	3 (3–4)	0.539
9, median (IQR)	4 (3–4)	4 (3–4)	3 (3–4)	0.512
Dementia, median (IQR)	4 (2–4)	4 (2–4)	3 (2–4)	0.539

Abbreviation: IQR: 25‐75th interquartile range.

**FIGURE 2:**
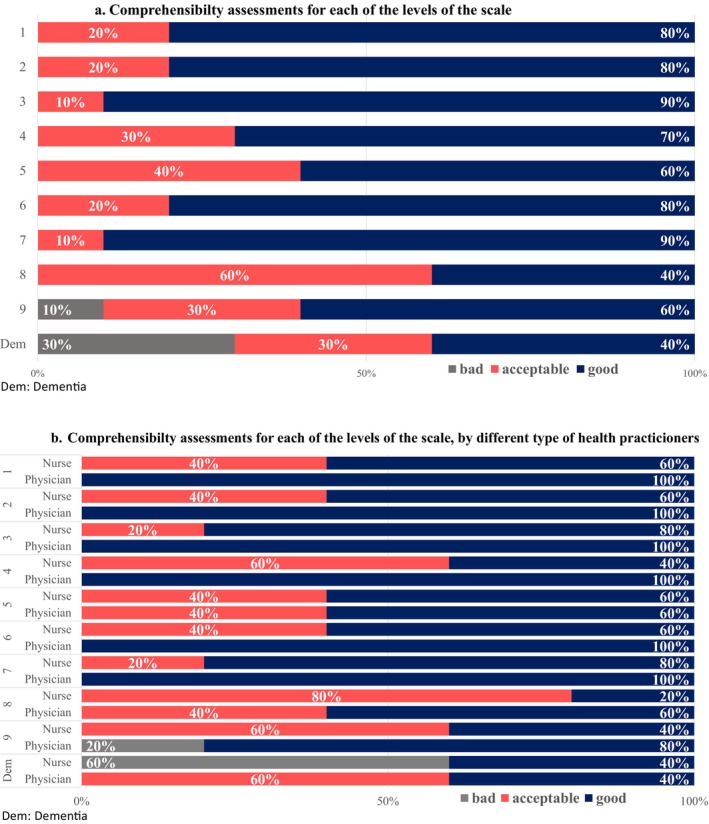
(a) Comprehensibility assessments for each of the levels of the scale. (b) Comprehensibility assessments for each of the levels of the scale, by different types of health practitioners.

Regarding the comprehensibility of the definitions for each level, only level 9 (rated poorly by one physician) and the assessment of people with dementia (rated poorly by 60% of nurses) received negative evaluations (Figure 2a,b). The confusion stemmed from how to assess these dementia patients or which frailty level to include them in.

Feedback from professionals resulted in minimal changes to the wording of some items, primarily in the section on assessing people with dementia (Table 2), yielding the final version of the CFS‐Es.

**TABLE 2:** Clinical Frailty Scale (CFS) and Clinical Frailty Scale‐España (CFS‐Es).

Level		Original definition	Spanish definición
1.	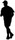	Very fit: People who are robust, active, energetic, and motivated. These commonly exercise regularly. They are among the fittest for their age.	Muy en forma: Personas que están fuertes, activas, enérgicas y motivadas. Son personas que suelen practicar ejercicio con regularidad. Son los que más en forma están para su edad.
2.	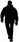	Well: People who have no active disease symptoms but are less fit than category 1. Often, they exercise or are very active occasionally, e.g., seasonally	En forma: Personas que no tienen síntomas de enfermedad activa, pero están menos en forma que las de la categoría 1. Suelen practicar ejercicio o son muy activas de forma esporádica. Por ejemplo, según la estación del año
3.	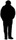	Managing well: People whose medical problems are well controlled but are not regularly active beyond routine walking	En buen estado: Personas cuyos problemas médicos están bien controlados, pero que no practican actividad física de forma regular más allá de los paseos habituales
4.	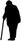	Vulnerable: While not dependent on others for daily help, symptoms often limit activities. A common complaint is being ‘slowed up’ and/or being tired during the day	Vulnerable: Personas no dependientes para actividades de la vida diaria, pero a menudo los síntomas limitan algunas actividades. Suelen quejarse de “ser lento” y/o estar cansado durante el día
5.	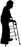	Mildly frail: These people often have more evident slowing, and need help in high order IADLs (finances, transportation, heaving housework, medications). Typically, mild frailty progressively impairs shopping, walking outside alone, meal preparation, and housework	Fragilidad leve: Personas que a menudo tienen un enlentecimiento más evidente y necesitan ayuda en actividades instrumentales de la vida diaria (economía, transporte, labores domésticas que requieren esfuerzo, medicación). Por lo general, la fragilidad leve incapacita progresivamente para salir solos de compras o a pasear, hacer la comida y las tareas domésticas
6.	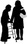	Moderately frail: People need help with all outside activities and with keeping house. Inside, they often have problems with stairs and need help with bathing and might need minimal assistance (cuing, standby) with dressing	Fragilidad moderada: Personas que necesitan ayuda en todas las actividades realizadas fuera de casa y las tareas domésticas. En casa, a menudo tienen dificultad con las escaleras, necesitan ayuda para bañarse y podrían necesitar asistencia mínima (estimulación, acompañamiento) para vestirse
7.	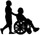	Severely frail: Completely dependent on personal care, from whatever cause (physical or cognitive). Even so, they seem stable and not at high risk of dying (within 6 months)	Fragilidad grave: Personas completamente dependientes para el cuidado personal, por cualquier causa (física o cognitiva). Aun así, parecen estables y sin gran riesgo de fallecer en los siguientes 6 meses
8.	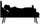	Very severely frail: Completely dependent, approaching the end of life. Typically, they could not recover even from a minor illness	Fragilidad muy grave: Personas totalmente dependientes y acercándose al final de la vida. En general, no podrían recuperarse ni de una enfermedad leve
9.	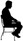	Terminally ill: Approaching the end of life. This category applies to people with a life expectancy < 6 months, who are not otherwise evidently frail	Enfermo terminal: Llegando al final de la vida. Esta categoría es para personas con esperanza de vida menor de 6 meses, tengan o no tengan signos evidentes de fragilidad
		Scoring frailty in people with dementia: The degree of frailty corresponds to the degree of dementia. Common symptoms in mild dementia include forgetting the details of a recent event, though still remembering the event itself, repeating the same question/story, and social withdrawal In moderate dementia, recent memory is significantly impaired, even though they seemingly can remember their past life events well. They can do personal care with prompting In severe dementia, they cannot do personal care without help	Puntuación de fragilidad en personas con demencia: Todo paciente con demencia se considera un paciente frágil y el grado de fragilidad se corresponde con el grado de demencia. Demencia leve (5. fragilidad leve): síntomas comunes en demencia leve incluyen olvidar detalles de acontecimientos recientes, aunque recuerden el acontecimiento en sí, repetir la misma pregunta/historia y aislamiento social Demencia moderada (6. fragilidad moderada): la memoria reciente está muy deteriorada, aunque parezca que recuerdan bien los acontecimientos del pasado. Con indicaciones, pueden realizar solos sus cuidados personales Demencia grave (7. fragilidad grave): los cuidados personales no son posibles sin ayuda

### 4.3 Concordance and Correlation of CFS‐Es With FRAIL‐Es

Between January 2020 and November 2023, frailty was assessed with CFS‐Es and FRAIL‐Es in 212 patients. Of these, 41% were women, distributed across age groups: < 50 years: 12.7%, 50–65 years: 23.1%, > 65 years: 64.2%.

The prevalence of frailty at admission for the 212 patients was 17.5% with CFS‐Es and 24.5% with FRAIL‐Es. The median (IQR) scores were 3 for CFS‐Es (3–4) and 1 for FRAIL‐Es (0–2). Both scales showed higher frailty prevalence in women and those over 65 years (Table 3).

**TABLE 3:** Descriptive analysis of patients' frailty at admission.

	Sex				Age			
	All *N* = 212	Women *N* = 87	Men *N* = 125	*p*	< 50 *N* = 27	50–65 *N* = 49	> 65 *N* = 136	*p*
CFS‐Es, patients, *n* (%)								
Nonfrail (1–3)	111 (52.4)	37 (42.5)	74 (59.2)	0.055	19 (70.4)	31 (63.3)	61 (44.9)	0.055
Vulnerable (4)	64 (30.2)	31 (35.6)	33 (26.4)		6 (22.2)	11 (22.4)	47 (34.6)	
Frail (5–9)	37 (17.5)	19 (21.8)	18 (14.4)		2 (7.4)	7 (14.3)	28 (20.6)	
FRAIL‐Es, patients, *n* (%)								
Nonfrail (0)	69 (32.5)	23 (26.4)	46 (36.8)	0.284	15 (55.6)	20 (40.8)	34 (25.0)	**0.017**
Prefrail (1–2)	91 (42.9)	41 (47.1)	50 (40.0)		7 (25.9)	20 (40.8)	64 (47.1)	
Frail (3–5)	52 (24.5)	23 (26.4)	29 (23.2)		5 (18.5)	9 (18.4)	38 (27.9)	

Abbreviations: CFS‐Es: Clinical Frailty Scale‐España; FRAIL‐Es: FRAIL‐España.

A total of 724 pairs of assessments were conducted at different time‐points: 212 at admission, and 162 at 3 months, 142 at 6 months, 114 at 9 months, and 94 at 12 months after hospital discharge. Out of the 724 assessments, 35.1% (*n* = 254) were classified as frailty using CFS‐Es and FRAIL‐Es, respectively (see Table 4).

**TABLE 4:** Descriptive analysis of all frailty assessments (baseline and at 3, 6, 9 and 12 months post‐hospital discharge).

CFS‐Es *n* = 724		FRAIL‐Es, *n* = 724	
1, Assessments, *n* (%) 2, Assessments, *n* (%) 3, Assessments, *n* (%)	23 (3.2) 58 (8.0) 183 (25.3)	0, Assessments, *n* (%)	247 (34.1)
4, Assessments, *n* (%)	206 (28.5)	1, Assessments, *n* (%) 2, Assessments, *n* (%)	152 (21.0) 148 (20.4)
5, Assessments, *n* (%) 6 Assessments, *n* (%) 7, Assessments, *n* (%) 8, Assessments, *n* (%) 9, Assessments, *n* (%)	118 (16.3) 76 (10.5) 43 (5.9) 16 (2.2) 1 (0.1)	3, Assessments, *n* (%) 4, Assessments, *n* (%) 5, Assessments, *n* (%)	130 (18.0) 42 (5.8) 5 (0.7)

Abbreviations: CFS‐Es: Clinical Frailty Scale‐España; FRAIL‐Es: FRAIL‐España.

The highest concordance (575 out of 724 assessments [79.4%]) was achieved by comparing two groups (non‐frail and frail), considering patients with CFS 4 as non‐frail (Table 5). In this scenario, 5% of non‐frail assessments with CFS‐Es (CFS‐Es 1–4) were classified as frailty with FRAIL‐Es (FRAIL‐Es 3–5), and 15.6% of frailty assessments with CFS‐Es (CFS‐Es 5–9) were categorised as non‐frailty with FRAIL‐Es (FRAIL‐Es 0–2) (Table 5).

**TABLE 5:** Concordance between CFS‐Es and FRAIL‐Es across all assessments (*n* = 724).

		Agreement, *n* (%)	CFS‐Es < FRAIL‐Es, *n* (%)	CFS‐Es > FRAIL‐Es, *n* (%)	Kappa (IC95%)
FRAIL‐Es (1a)	CFS‐Es (2a)	444 (61.3)	116 (16.0)	164 (22.7)	0.424 (0.374–0.475)
FRAIL‐Es (1b)	CFS‐Es (2b)	575 (79.4)	36 (5.0)	113 (15.6)	0.514 (0.444–0.585)
FRAIL‐Es (1b)	CFS‐Es (2c)	439 (60.6)	1 (0.1)	284 (39.2)	0.308 (0.255–0.361)

*Note:* (1a) 0: Not frail, 1–2: Pre‐frail, 3–5: Frail; (1b) 0–2: Not frail, 3–5: Frail. (2a) 1–3: Not frail, 4: Vulnerable, 5–9: Frail; (2b) 1–4: Not frail, 5–9: Frail; (2c) 1–3: Not frail, 4–9: Frail.

Abbreviations: CFS‐Es: Clinical Frailty Scale‐España; CI: Confidence Interval; FRAIL‐Es: FRAIL‐España.

**FIGURE 3:**
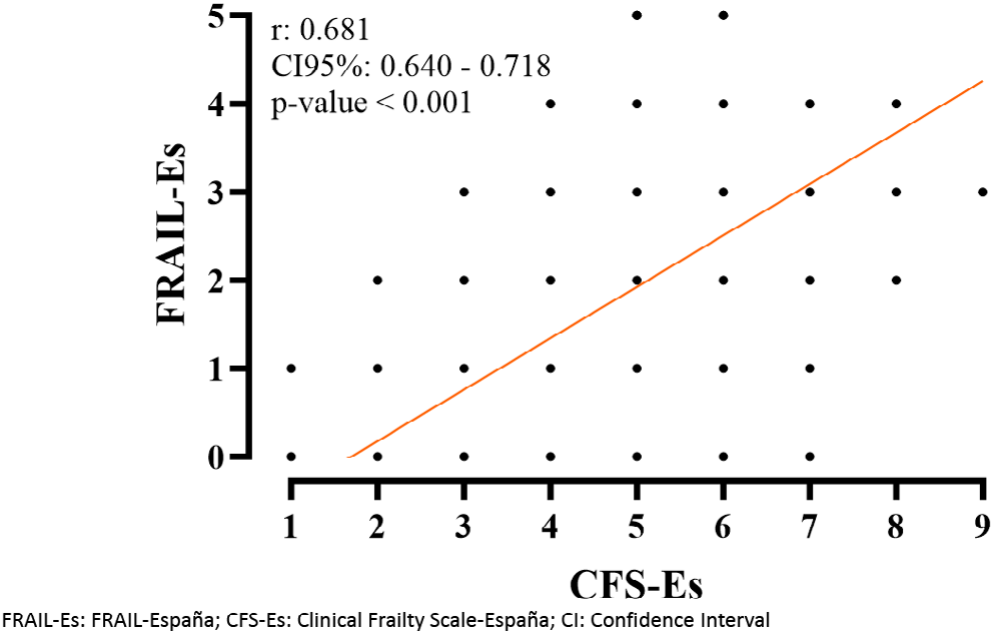
Correlation between CFS‐Es and FRAIL‐Es.

The correlation between CFS‐Es and FRAIL‐Es was positive and strong (*r* [95% CI]: 0.681 [0.640–0.718]) (Figure 3).

## 5 Discussion

The adaptation of the CFS has been achieved with minimal changes compared to the original scale, consistent with trends observed in adaptations to other languages (Chou et al. 2022; Nissen et al. 2020), except in the section concerning people with dementia. Here, the structure has been modified to clarify the assessment of frailty levels. During the pilot phase, professionals found levels 8 and 9 similar, as both groups of patients approach the end of life, although those in level 9 are not entirely dependent (Rockwood and Theou 2020). Evaluating frailty upon admission to the intensive care unit (ICU) allows for developing specific care plans based on prior frailty, thus avoiding further deterioration in more frail patients during ICU stay.

It is crucial to assess frailty based on the baseline situation before admission, as terminal illnesses may be diagnosed during hospitalisation, which was unknown at the time of assessment. Evaluators considered it less relevant to change the previous assessment when diagnosing terminal illnesses, placing them in level 9. This is because care plans developed based on their previous physical activity or dependence would not be altered by a prognosis of less than 6 months of life.

Although the scale adaptation was carried out in 2019, and the authors published version 2.0 in 2020 (Rockwood and Theou 2020), the definitions in the Spanish version do not significantly differ from either of the two original versions. Even the text could be considered more akin to version 2.0. While in the 2007 version, people in level 4 are categorised as ‘vulnerable’ (which is maintained in the Spanish version), in version 2.0 (Rockwood and Theou 2020), they are described as ‘living with very mild frailty’. However, the definition of this level does not vary, so the Spanish version could also be considered an adaptation of CFS 2.0.

The prevalence in our patient cohort, assessed upon admission with CFS‐Es, is lower than that reported in systematic reviews of studies with critical patients (30%–39.4%) (Chan et al. 2022; Muscedere et al. 2017). It is essential to note that most studies included in these reviews focus on older patients. In this regard, the found prevalence is similar to that in studies from these reviews and other more recent studies conducted with critical patients aged 18 or older (Darvall et al. 2019; Fisher et al. 2015; Georgiou et al. 2023; Low et al. 2022; Remelli et al. 2023; Subramaniam et al. 2022), where prevalences between 13% and 18.9% are observed. The higher prevalence observed in women, both with CFS‐Es and FRAIL‐Es, has been previously reported, as well as the higher prevalence of frailty in those over 65 years (Church et al. 2020; Gordon et al. 2017).

Despite the scales evaluating frailty through different aspects of patients' baseline situations, we have observed moderate concordance in frailty assessment with CFS‐Es and FRAIL‐Es. Although numerous scales assess frailty using different approaches and robust theoretical frameworks, there is no consensus on using a particular approach (De Biasio et al. 2020).

The CFS assesses physical activity and dependence, while the FRAIL scale evaluates frailty through five domains, including physical activity, comorbidities and unintentional weight loss. The different ways of assessing frailty with these scales may have contributed to not obtaining more concordance between them. Another factor could be the subjectivity in the application of CFS‐Es, as noted by some evaluators in the pilot test, due to the lack of closed‐ended questions, an observation previously made by other authors (De Biasio et al. 2020). However, the observed correlation is strong, similar to that obtained between the Korean versions of CFS‐K and FRAIL‐K (*R* = 0.8053) (Ko et al. 2021).

### 5.1 Limitations of the Study

Assessing the criterion validity of CFS‐Es is challenging due to the absence of a gold‐standard scale in frailty assessment, which prevents the evaluation of CFS‐Es sensitivity and specificity. However, the strong correlation and moderate concordance with FRAIL‐Es suggest the validity of both scales. Although it will be necessary to examine other psychometric properties in future research, the joint evaluation of CFS‐Es and FRAIL‐Es could facilitate more accurate detection of the most vulnerable patients and the planning of more specific care plans.

Another significant limitation of the study is the need to assess frailty through family members rather than directly through the patient due to the latter's inability to communicate effectively in some instances. Family members may not be fully aware of their relative's condition, but both scales have been implemented in the same way at the same time by the same evaluator.

### 5.2 Implications for Practice

Following a transcultural adaptation process, we have obtained the Spanish version of CFS for application in adult critical patients (≥ 18 years) admitted to intensive care. Frailty is a construct between normal physiological aging changes and the final state of disability (Abellan van Kan, Rolland, Morley, et al. 2008). However, it is important to note that physiological age, not just chronological age, is challenging to determine. The same deficits associated with frailty in elderly patients can be observed in critical patients, regardless of age and disease severity (McDermid, Stelfox, and Bagshaw 2011). Therefore, assessing frailty in a patient admitted to intensive care can be helpful for better planning the care of frail patients. This tool can provide valuable information for developing specific care plans tailored to each patient's needs, thus contributing to more precise and personalised care in the intensive care setting.

## 6 Conclusions

The Spanish version of the CFS has proven to be a simple and easy‐to‐implement tool in adult critical patients, effectively used by nurses and physicians. Although non‐significant differences were observed between both professions, it is important to note that the scale involves a certain degree of subjectivity due to the absence of closed‐ended responses.

The prevalence of frailty varies depending on the scales used. However, we have shown an optimal agreement and correlation between CFS‐Es and FRAIL‐ES, with patients at level 4 of the FRAIL‐ES being considered vulnerable but not frail.

This adaptation has allowed for a more accurate assessment of frailty in critical patients, offering a valuable approach to identifying those who may require special attention and more specific care plans. These findings support the utility of CFS‐Es in the intensive care context, facilitating clinical decision‐making and improving patient‐centred care.

It is important to emphasise the active participation of intensive care nurses and physicians in adapting the CFS‐Es and assessing their concordance and correlation with the FRAIL‐Es. Evaluating frailty should be an interprofessional and collaborative effort, as the patient's frailty level can impact both nursing care and medical treatment.

## Author Contributions

Conceptualisation: S.A.‐R. Methodology: S.A.‐R., M.N.M.‐T., M.R.‐T. Software: S.A.‐R. Validation: S.A.‐R., M.N.M.‐T., F.F.‐V., C.A.‐V., I.J.T.‐V., M.M.S.‐S., R.S.‐I., L.O.‐L., S.L.‐C., M.C.‐L., R.J.‐C., V.L.‐L., E.I.S.‐M., L.F.C.R.‐R., M.J.F.‐M., I.C.‐P., R.P.‐P., A.H.‐G., A.L.‐G.A., F.G.‐V., A.R.‐M., M.V.‐C., G.V.‐G., D.M., R.H.‐H., M.R.‐T. Formal analysis: S.A.‐R., I.J.T.‐V. Investigation: S.A.‐R., M.N.M.‐T., F.F.‐V., C.A.‐V., I.J.T.‐V., M.M.S.‐S., R.S.‐I., L.O.‐L., S.L.‐C., M.C.‐L., R.J.‐C., V.L.‐L., E.I.S.‐M., L.F.C.R.‐R., M.J.F.‐M., I.C.‐P., R.P.‐P., A.H.‐G., A.L.‐G.A., F.G.‐V., A.R.‐M., M.V.‐C., G.V.‐G., D.M., R.H.‐H., M.R.‐T. Resources: S.A.‐R., F.F.‐V., C.A.‐V., I.J.T.‐V., M.M.S.‐S., R.S.‐I., L.O.‐L., S.L.‐C., M.C.‐L., R.J.‐C., V.L.‐L., E.I.S.‐M., L.F.C.R.‐R., M.J.F.‐M., I.C.‐P., R.P.‐P., A.H.‐G., A.L.‐G.A., F.G.‐V., A.R.‐M., M.V.‐C., G.V.‐G., D.M. Data Curation: S.A.‐R. Writing – Original Draft: S.A.‐R. Writing – Review and Editing: S.A.‐R., M.N.M.‐T., F.F.‐V., C.A.‐V., I.J.T.‐V., M.M.S.‐S., R.S.‐I., L.O.‐L., S.L.‐C., M.C.‐L., R.J.‐C., V.L.‐L., E.I.S.‐M., L.F.C.R.‐R., M.J.F.‐M., I.C.‐P., R.P.‐P., A.H.‐G., A.L.‐G.A., F.G.‐V., A.R.‐M., M.V.‐C., G.V.‐G., D.M., R.H.‐H., M.R.‐T. Visualisation: S.A.‐R., F.F.‐V., R.H.‐H., M.R.‐T. Supervision: S.A.‐R. Project administration: S.A.‐R. Funding acquisition: S.A.‐R. Critical revisions for important intellectual content: All the authors reviewed the final manuscript before submitting it for publication.

## Ethics Statement

The study protocol was reviewed and approved by the Ethics Committee for Drug Research (CEIm) of the Hospital Universitario de Getafe (IRB number: CEIm2019/42).

## Conflicts of Interest

The authors declare no conflicts of interest.

## Supporting information


Data S1.

## Data Availability

The data that support the findings of this study are available from the corresponding author upon reasonable request.
