# Correction to ‘Cross‐Cultural Adaptation of the Clinical Frailty Scale for Critically Ill Patients in Spain and Concurrent Validity With FRAIL‐Es’

**DOI:** 10.1002/nop2.70683

**Published:** 2026-07-31

Arias‐Rivera S , Moro‐Tejedor MN , Frutos‐Vivar F , et al. 2025. “Cross‐Cultural Adaptation of the Clinical Frailty Scale for Critically Ill Patients in Spain and Concurrent Validity With FRAIL‐Es.” Nursing Open 12(2):e70064. 10.1002/nop2.70064.39962771 PMC11832588

In the Funding information the text ‘The present work was supported by the Ministry of Economy and Competitiveness ISCIII‐FIS grant PI20/01231’ was incorrect. This should have read: ‘Funded by grant from the Spanish Ministry of Economy, Industry and Competitiveness, co‐financed by the European Regional Development Funds (Instituto de Salud Carlos III, PI20/01231)’.

We apologize for this error.

