# Supplementary material for: Cross‐Cultural Adaptation of the Clinical Frailty Scale for Critically Ill Patients in Spain and Concurrent Validity With FRAIL‐Es
Source: Nurs Open. 2025 Feb 17;12(2):e70064. doi: 10.1002/nop2.70064 (PMC11832588; doi:10.1002/nop2.70064)
Supplement: Supplementary file 1 — Data S1. [file NOP2-12-e70064-s001.pdf]

DALHOUSIE UNIVERSITY

GERIATRIC MEDICINE RESEARCH

FOR OFFICE USE ONLY
PF#: 20211223-04

PERMISSION TO USE THE CLINICAL FRAILTY SCALE (CFS)

The undersigned is granted permission to use, reproduce and distribute the Clinical Frailty Scale (CFS), developed by Dr. Kenneth Rockwood, in the format attached[1] for educational purposes and for non-commercially funded research and/or quality assurance projects. The CFS must be administered free of charge to patients and/or study participants. A formal Licensing Agreement is required for research funded by any commercial entity or pharma and, in some cases, for use in routine clinical care. The copyright holder reserves the right to prospectively follow-up at any time to determine whether use of the CFS meets the conditions described above. Reselling of the CFS or other commercial development without a license agreement is prohibited by copyright. The undersigned, their delegates and affiliated organization(s) agree that they will not claim ownership rights to the CFS, or any derivative, including translations, compilation, sequel or series. Nothing in this Agreement shall give the undersigned any right, title, or interest in the CFS other than the right to use in accordance with this Agreement. The CFS will not be modified unless explicit permission is granted.

**USER INFORMATION:**	
Full Name:	Susana Arias-Rivera
Position/Title:	Nursing Research
Institution/Organization:	Hospital Universitario de Getafe, Madrid (España)
Mailing Address:	Carretera de Toledo, Km. 12,5. 28905 Getafe (Madrid), Spain
Telephone:	+34[redacted]
Email:	susana.arias@salud.madrid.org
Type of organization:	☐ For-profit ☐ Not-for-profit ☒ Other, *please specify:* Public healthcare

INTENDED USE (Select all that apply):

☒ Reprint *Provide publication details:* Adaptation to Spanish (Spain) and validation in critical ill patients

☒ Research study/clinical trial *Expected duration of study:* Start date 2019 End date 2023
Describe use in study: Implementation of the scale, to patients with inclusion criteria, upon admission to the intensive unit (baseline), and 3, 6, 9 and 12 months after hospital discharge
Is research sponsored or funded by pharma or industry? ☐ Y ☒ N
***If yes**, please specify:*

☐ Routine clinical care
Will the CFS be incorporated into an electronic medical/health record (EMR)? ☐ Y ☒ N
***If yes**, please identify whether an EMR company is involved (e.g., Epic, Meditech):*

☐ Other *Specify:*

Are you planning to translate the CFS? ☒ Y ☐ N *If yes, specify language(s):* Spanish (Spain)
We request editable (e.g. MSWord) copies of all translations. We do not independently verify or validate translations.

Are you planning any commercial development that would incorporate the CFS?
☐ Y ☒ N *If yes, please specify:*

By your signature below, you attest that you understand the conditions under which permission is granted.

Signature:	ARIAS RIVERA, SUSANA (FIRMA) Firmado digitalmente por ARIAS RIVERA, SUSANA (FIRMA) Fecha: 2021.12.16 08:37:37 +01'00'
Date:	16.12.2021

Send completed, signed form by fax or email to:	**Geriatric Medicine Research**<br>1421-5955 Veterans' Memorial Lane, Halifax, NS B3H 2E1 Canada<br>Fax: 1-902-473-1050 | Email: gmru@dal.ca

Approved by: [signature] Date: 2021/12/23
Developer or Designated Authority

[1]A copy of the CFS will be sent to the user upon review and approval of this permission form. Valid only when signed by all parties.
